# Prediction of Speech-Correcting Surgery in Patients With a Cleft Palate After Primary Palatoplasty: A Logistic Regression Model

**DOI:** 10.1177/10556656251401507

**Published:** 2025-12-02

**Authors:** Lieke Hofman, Kevin Jenniskens, Harm Winters, Aebele B. Mink van der Molen, Emma C. Paes

**Affiliations:** 1 Department of Pediatric Plastic Surgery, 89098 Wilhelmina Children's Hospital, University Medical Center Utrecht, Utrecht University, Utrecht, The Netherlands; 2 Julius Center for Health Sciences and Primary Care, 168086 University Medical Center Utrecht, Utrecht University, Utrecht, The Netherlands; 3 Cochrane Netherlands, 8124 University Medical Center Utrecht, Utrecht University, Utrecht, The Netherlands

**Keywords:** cleft palate, palatoplasty, speech correcting surgery, prediction model

## Abstract

**Objective:**

To identify predictors of speech-correcting surgery after primary palatoplasty in patients with cleft palate with or without cleft lip (CP ± L) and to develop pre- and postoperative prediction models.

**Design:**

Retrospective cohort study.

**Setting:**

Wilhelmina Children's Hospital, Utrecht, the Netherlands.

**Patients, Participants:**

A total of 239 patients with CP ± L who underwent primary palatoplasty between 2008 and 2017 and completed standardized speech assessment at age 5.

**Interventions:**

Straight-line palatoplasty with intravelar veloplasty (Sommerlad) within the first year of life.

**Main Outcome Measure(s):**

Likelihood of speech-correcting surgery after primary palatoplasty. Potential predictors included cleft type, cleft width, age at palatoplasty, associated syndromes, and postoperative complications such as palatal dehiscence and oronasal fistula. Logistic regression models were developed using pre- and postoperative variables. Model performance was assessed by AUROC, calibration, Brier score, and *R*².

**Results:**

Of 239 patients, 49% required speech-correcting surgery. In the preoperative model, cleft width and presence of syndromes were significant predictors, showing moderate discrimination (AUROC: 0.694; 95% CI: 0.620–0.759) and good calibration. Adding oronasal fistula in the postoperative model minimally improved performance (AUROC: 0.697; 95% CI: 0.621–0.764).

**Conclusions:**

A clinically applicable model was developed to predict the likelihood of speech-correcting surgery following primary palatoplasty. Wide clefts, the presence of syndromes, and oronasal fistula were identified as key predictors.

## Introduction

Cleft palate with or without cleft lip (CL ± P) is a congenital craniofacial anomaly that presents a complex treatment challenge.^1^ The primary goal of palatoplasty is to restore normal palatal function, ensuring adequate velopharyngeal closure to support feeding, hearing and speech development.^2^ However, velopharyngeal insufficiency (VPI) remains a common complication, affecting up to 47% of patients with CP ± L and is associated with persistent speech disorders. These include obligatory errors such as hypernasality and nasal emission, which result from structural deficits, and compensatory errors such as articulation errors that develop in response of these deficits.^3–5^ While compensatory errors can be treated with speech therapy alone, obligatory errors often necessitate additional speech-correcting surgery.^6^

Comparing speech outcomes between studies remains challenging and a considerable variation exists in VPI prevalence and rates of speech-correcting surgery, reflecting differences in patient cohorts, surgical protocols (techniques, timing), surgeon experience, speech assessment methods, and center-specific thresholds for performing speech-correcting surgery.^3^ Insufficient velopharyngeal function and associated speech disorders may be related to patient factors such as cleft extent and width, presence of additional syndromes and the age at which palatoplasty is performed.^3^ Additionally, postoperative complications such as infection, impaired wound healing or oronasal fistula, can lead to VPI due to disrupted healing.^7,8^ Moreover, the decision to perform a speech-correcting surgery may vary across countries, as mild residual VPI causing hypernasality can be more perceptible in one language compared to another.^7^

At our center, primary palatoplasty is performed before 12 months of age using a 2-stage procedure: the hard palate is closed during lip repair using a vomer flap, followed by soft palate closure using a straight-line technique with intravelar veloplasty, as described by Sommerlad.^9^ Parents are often interested in the success rate of the primary surgery and the probability of requiring additional speech-correcting surgery in the future.^10^ Therefore, accurate preoperative consultation is essential to set realistic expectations. Yet, predicting which patients will need speech-correcting surgery to address VPI remains challenging.

This study aims to identify key predictors and develop a model for predicting speech-correcting surgery following palatoplasty. Prediction models using information available before and after primary palatoplasty were developed separately.

## Materials and Methods

This manuscript was written in accordance with the Transparent Reporting of a multivariable prediction model for Individual Prognosis Or Diagnosis (TRIPOD + AI) guidelines.^11^

### Data Collection

A retrospective cohort study was conducted involving patients with CP ± L who underwent primary palatoplasty at the Wilhelmina Children's Hospital in Utrecht, the Netherlands (2008‒2017). Both syndromic and nonsyndromic patients with CP ± L patients (i.e., those with or without a genetic disorder, clinical syndromic diagnosis or additional (craniofacial) anomalies) were included. Ethical approval was obtained from the institutional review board of the hospital (No: 22U-0168). Patients were eligible for inclusion if they underwent Sommerlad palatoplasty and underwent speech assessment by a speech-language pathologists at the age of 5. Exclusion criteria were submucous cleft palate, primary palatoplasty elsewhere, adoption, incomplete medical records or lost to follow-up. A minimum follow-up of 5 years was required to ensure reliable speech assessment.

### Surgical Protocol

At our center, the hard and soft palates are closed between 6 and 12 months of age. In patients with a complete cleft of the lip, alveolus and palate, the hard palate is closed concurrently with lip repair using a vomer flap, followed by closure of the soft palate in a second procedure using the Sommerlad technique.^12^ In patients with an isolated cleft palate, the hard and soft palate are closed simultaneously within the first year of life. Using the Sommerlad technique, the soft cleft palate is restored by performing a straight-line palatoplasty with intravelar veloplasty, under microscopic magnification.^9^ First, marginal cleft incisions are made from the hard palate to the uvula. Then, bilateral mucosal flaps are raised and the nasal layer and the velar musculature are freed from the palatal shelves. The nasal mucosa is closed in the midline, and the levator veli palatini muscle is released from its abnormal insertion, mobilized laterally as far as possible until the muscle enters the levator tunnel and repositioned and repaired in the midline to establish an effective muscular sling. Finally, the oral mucosal layer is closed, using relaxing oral mucosal incisions if necessary.^9^

### Outcome

The primary aim of this study is to identify key predictors of speech-correcting surgery to address VPI and develop a predictive model to estimate the individual risk of requiring additional speech-correcting surgery after primary palatoplasty.

The advice to perform speech-correcting surgery was determined by multidisciplinary consensus including the plastic surgeon, ear, nose and throat surgeon, and speech-language pathologist based on speech assessment and supported by either nasoendoscopy, videofluoroscopy or both. Speech variables such as hypernasality, nasal air emission, and poor intelligibility were used as indicators of VPI. The speech-language pathologist evaluated speech intelligibility based on spontaneous speech using a 5-point scale ranging from normal to unintelligible speech and differentiated obligatory errors, caused by structural deficits, from compensatory errors. When VPI was confirmed on nasoendoscopy or videofluoroscopy and obligatory errors were present that were unlikely to improve with further speech therapy, additional speech-correcting surgery was suggested. The ultimate decision to perform additional surgery was made in shared decision making with the patient and parents. Speech-correcting surgery was selected as the outcome measure, representing VPI that persisted despite intensive speech therapy. While VPI diagnosis can vary across assessment methods, expertise, and treatment centers, the decision to perform speech-correcting surgery consistently represents functionally significant impairment.

### Candidate Variables—Preoperative Model

Separate pre- and postoperative models were developed. Potential predictors for the preoperative model were cleft type, initial cleft width (measured in the operation theater before palatoplasty), age at which palatoplasty was performed, and the presence of additional syndromes. Variables were selected on their known or potential association with speech outcomes based on current literature, and expert opinion.^3^ Cleft type was classified before primary palatoplasty according to the Veau classification (Veau I: soft cleft palate; Veau II: hard and soft cleft palate; Veau III: unilateral cleft lip/alveolus/palate; Veau IV: bilateral cleft lip/alveolus/palate).^13^ Cleft width was measured in millimeters using a Castroviejo caliper at the junction of the hard and soft palate before primary palatoplasty. Age was classified as age in months during soft palatoplasty. Having an additional syndrome was classified as present or absent.

### Candidate Variables—Postoperative Model

The postoperative model additionally included palatal dehiscence and oronasal fistula. Palatal dehiscence was defined as a 1- or 2-layer opening in the palate with spontaneous closure in the first 3 months following surgery. Oronasal fistula was defined as a persistent 2-layer (nasal and oral) opening in the palate following 3 months after surgery. Both were confirmed by the plastic surgeon during routine follow-up at 6 and 12 weeks postoperatively. Fistulas were addressed prior to or at the time of speech-correcting surgery. In the latter group, the indication for surgery was insufficient velopharyngeal function due to a short palate, which was evaluated using nasoendoscopy or videofluoroscopy, rather than air leakage through the fistula.

### Statistical Analysis

All categorical variables are presented as frequencies and percentages. Continuous variables are expressed as mean ± standard deviation (SD) or median (interquartile range). Variables with a skewed distribution were transformed using the natural logarithm prior to model inclusion.

Logistic regression models using pre- and postoperative data were constructed. Selection of informative variables in the parsimonious model was performed using backward selection with *p*-value <.157 [based on the Akaike information criterion (AIC)].^14^ Beta coefficient, standard error, Wald test *p*-value and odds ratio (OR) were reported for each predictor in the final model.

Model performance was assessed in terms of discrimination, calibration and overall performance. Discrimination was assessed using the area under the receiver operator characteristic (AUROC). Calibration plots were generated from bootstrapped samples (*n* = 1000) and performance was evaluated by comparing predicted probabilities from the model to the actual observed outcomes. Performance in terms of calibration was assessed by visual inspection of the calibration plot, calibration intercept and slope. The scaled Brier score, with 0 indicating a noninformative model and 1 a perfect model, and *R*^2^ were used as overall model performance measures.

Internal validation was performed by combining multiple imputation for missing values (*n* = 10 imputation datasets) with 5-fold cross-validation. Optimism-corrected performance measures, including 95% confidence intervals, were calculated.

Statistical analysis was performed using SPSS Statistics software function version 26.0 for baseline data, and R-studio for model development and internal validation.

## Results

### Study Population

The study included 239 patients with CP ± L, with a mean age of 10 years at postoperative follow-up (SD = 3.0, range = 5–16 years). The mean age at soft palatoplasty was 10 months (SD = 2.8, range = 3–22 months). Other relevant patient characteristics are summarized in Table 1. In 119 patients (49%), additional speech-correcting surgery was required. The mean age at which a speech-correcting surgery was performed was 5.3 years (SD = 1.6, range = 3–10 years).

**Table 1.** Patient Characteristics.

	Patients with SCS (%) (*N* = 119)	Patients without SCS (%) (*N* = 120)	Total (%) (*N* = 239)
Gender			
Male	74 (62)	54 (45)	128 (54)
Female	45 (38)	66 (55)	111 (46)
Cleft type			
Veau I	11 (9)	22 (18)	33 (14)
Veau II	37 (31)	48 (40)	85 (35)
Veau III	53 (45)	37 (31)	90 (38)
Veau IV	18 (15)	13 (11)	31 (13)
Initial cleft width (mm)^a^			
Mean (SD)	12 (3.2)	9.69 (2.9)	10.9 (3.3)
Median [range]	13 [3–22]	10 [3–16]	11 [3–22]
Age at soft palatoplasty (months)			
Mean (SD)	10.2 (2.9)	10.1 (2.9)	10 (2.8)
Median [range]	10 [3–18]	10 [5–22]	10 [3–22]
Syndrome	43 (36)	43 (36)	86 (36)
Palatal dehiscence	14 (12)	10 (8)	24 (10)
Oronasal fistula	30 (25)	13 (11)	43 (18)

SD = standard deviation; Veau I = soft cleft palate; Veau II = hard and soft cleft palate; Veau III = unilateral cleft lip/alveolus/palate; Veau IV = bilateral cleft lip/alveolus/palate.

a Data on cleft width was not available in 21 patients. SCS = speech-correcting surgery.

### Preoperative Model

The final preoperative model included cleft width and presence of syndromes, as they met the AIC threshold of <0.157 (*p* < .001 and *p* = .146) (Table 2).

**Table 2.** Final Pre- and Postoperative Model for Predicting the Risk of Speech-Correcting Surgery.

Variable	Estimate	SE	*P*-value	OR
Preoperative model				
Intercept	−2.929	0.598	−	−
Cleft width	0.254	0.049	<.001	1.29
Syndrome	0.443	0.304	.146	1.56
Postoperative model				
Intercept	−2.922	0.597	−	−
Cleft width	0.240	0.050	<.001	1.27
Syndrome	0.479	0.306	.118	1.61
Oronasal fistula	0.780	0.385	.044	2.18

Intercept: represents the log-odds of the baseline risk of needing additional speech-correcting surgery when all independent variables are absent. *P*-value according to the Wald test; OR = odds ratio.

**Figure 1.**
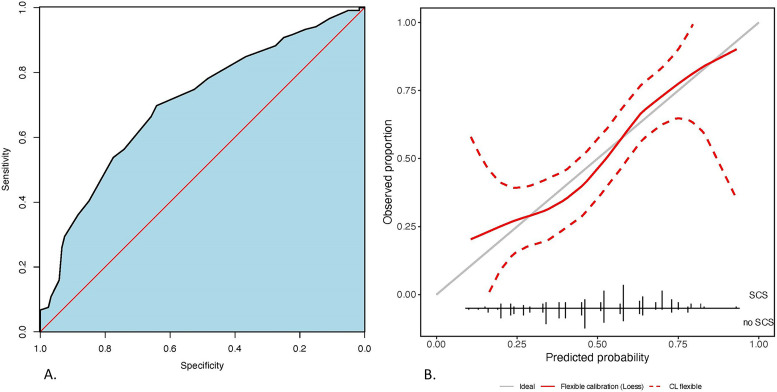
(a) Discriminatory performance of the preoperative model for speech-correcting surgery after primary palatoplasty. Blue area indicates the area under the ROC curve. Red line is the random chance reference line; (b) Calibration of the preoperative model for speech-correcting surgery after primary palatoplasty. Vertical solid black lines at the bottom indicate proportion of speech-correcting surgery/no speech-correcting surgery for a given predicted probability. Grey line is the reference line. Red line is the smoothed curve of the model. Dashed red line is 95% intervals.

The naïve AUROC of the preoperative model was 0.705 (0.639–0.770). After cross-validation, the optimism-corrected AUROC was 0.694 (95% CI: 0.620-0.759), indicating moderate discriminative ability (Figure 1(a)).

Figure 1(b) illustrates preoperative model calibration after internal validation. Visual inspection of the calibration plot indicates that there is some remaining uncertainty across the range of all predicted probabilities, however, the model provides well calibrated predicted probabilities on average. This is substantiated by the calibration intercept of −0.003, and calibration slope of 0.981 (Table 3).

**Table 3.** Performance of the Pre- and Postoperative Prediction Models for Speech-Correcting Surgery.

	Preoperative model	Postoperative model
AUC (95% CI)	0.694 (0.620−0.759)	0.697 (0.621−0.764)
Calibration intercept	−0.003	0.010
Calibration slope	0.981	0.933
Scaled Brier score	0.105	0.106
*R* ^2^	.168	.171

Overall performance is reflected by the scaled Brier score of 0.105 and *R*² value of 0.168. The latter indicates that the model explains 16.8% of the variation in need for speech-correcting surgery. While 16.8% is a modest level of explanation, it is common for this value to be relatively low, especially for complex or multifactorial outcomes.

### Postoperative Model

The postoperative model was expanded with palatal dehiscence and oronasal fistula as additional candidate variables. Besides cleft width and presence of syndromes, only oronasal fistula met the AIC threshold of <0.157 (*p* = .044) (Table 2).

**Figure 2.**
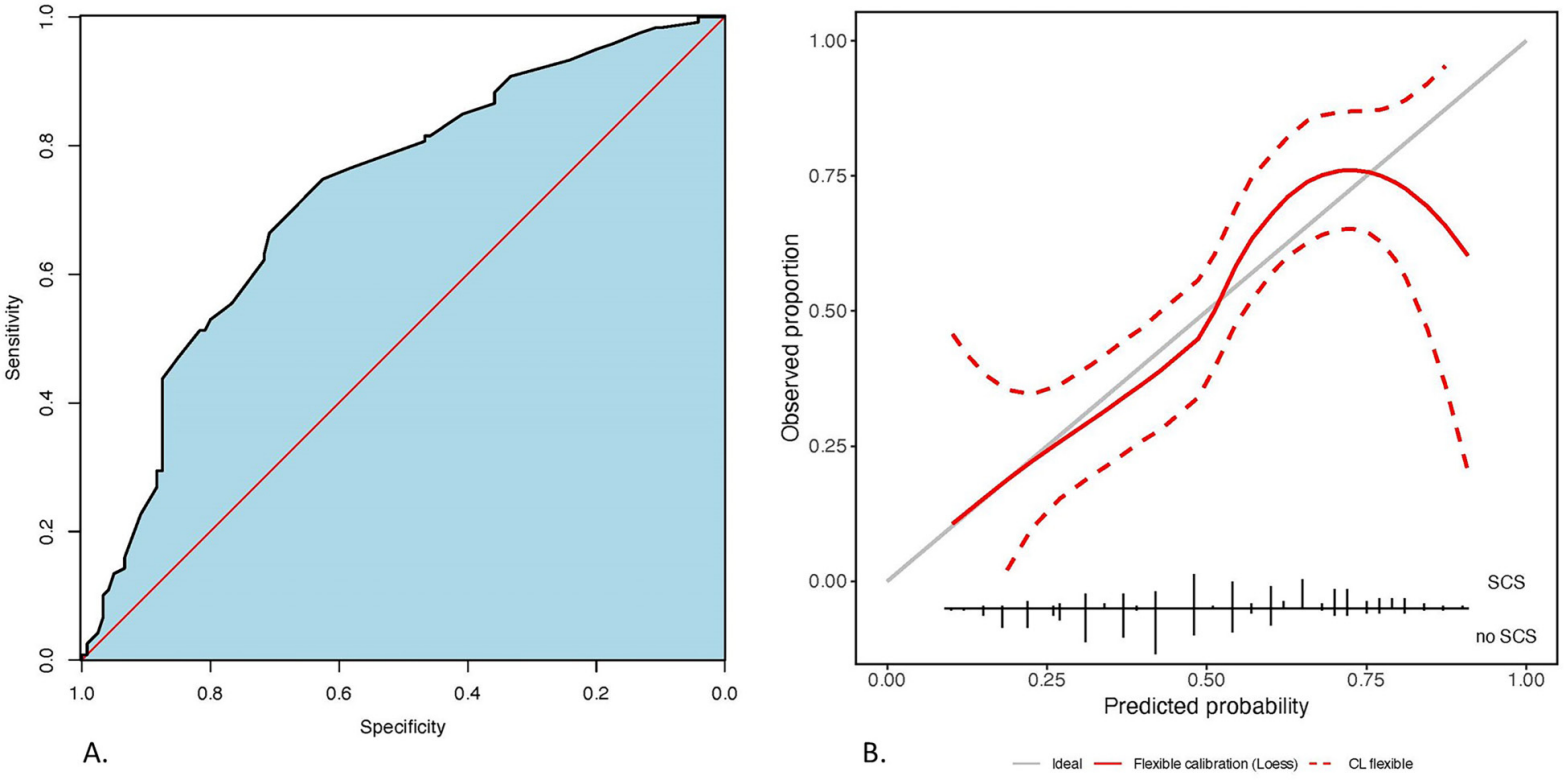
(a) Discriminatory performance of the postoperative model for speech-correcting surgery after primary palatoplasty. Blue area indicates the area under the ROC curve. Red line is the random chance reference line; (b) Calibration of the postoperative model for speech-correcting surgery after primary palatoplasty. Vertical solid black lines at the bottom indicate proportion of speech-correcting surgery/no speech-correcting surgery for a given predicted probability. Grey line is the reference line. Red line is the smoothed curve of the model. Dashed red line are 95% intervals.

The naïve AUROC for the postoperative model was 0.723 (0.659‒0.787). After cross-validation the optimism-corrected AUROC was 0.697 (95% CI: 0.621‒0.764) (Figure 2a).

Figure 2(b) illustrates the postoperative model calibration after internal validation. Visual inspection of the calibration plot indicates that there is some remaining uncertainty across the range of all predicted probabilities. The model provides well calibrated predicted probabilities on average, with slight underestimation of predicted probabilities in the range of 0.55‒0.75. The calibration intercept and slope were found to be 0.010 and 0.933 respectively (Table 3).

Overall performance according to the scaled Brier score was 0.106. The *R*^2^ of the postoperative was .171, indicating that this model explains 17.1% of the variation in the outcome.

**Figure 3.**
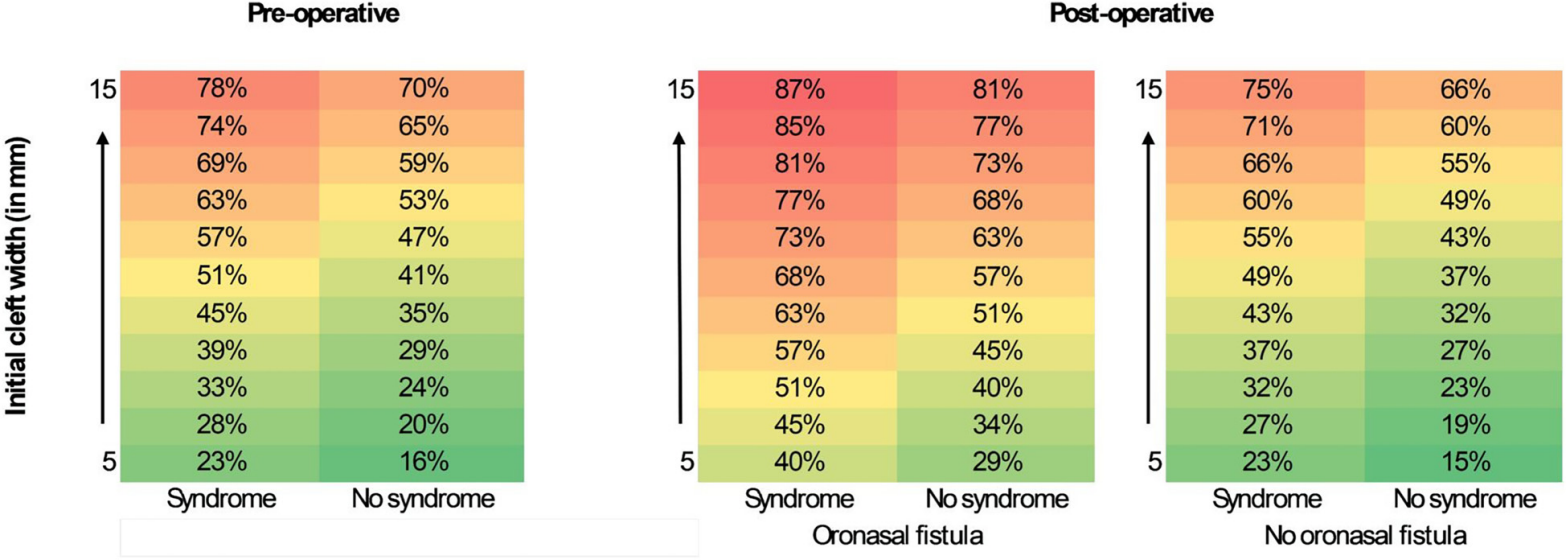
This heatmap illustrates the relationships between the different key predictors (wide cleft, syndrome and oronasal fistula) and the predicted probability of undergoing additional speech-correcting surgery. Each cell represents the predicted probability (%) of requiring speech-correcting surgery given a specific combination of predictor values. Color gradients indicate risk categories, with red representing the highest predicted risk and green the lowest.

To facilitate clinical implementation, predicted probabilities of speech-correcting surgery were translated into a heatmap based on combinations of key predictors identified in the final models (Figure 3). For example, in the preoperative model, a patient with a syndrome and a cleft width of 10 mm would have a 51% predicted probability of requiring speech-correcting surgery. In contrast, a patient without a syndrome and with a cleft width of 5 mm has a much lower risk (16%).

## Discussion

This study identified a wide cleft, the presence of syndromes, and oronasal fistula as predictors of speech-correcting surgery, with a wide cleft being the strongest predictor. In contrast, cleft type, age at which palatoplasty was performed and palatal dehiscence were not strongly associated with the need for speech-correcting surgery. The predictive variables were included into pre- and postoperative prediction models designed as clinical risk tools to support patient and parents consultation.

This study specifically focuses on predicting the need for speech-correcting surgery following primary palatoplasty, rather than describing cleft speech outcomes and VPI, which were addressed in our previous work.^12^ Only patients undergoing soft palate closure with the Sommerlad technique were included, with the hard palate previously closed during lip repair. This 2-stage approach, incorporating a vomer flap at lip closure, has been used at our center since 2008 to reduce cleft width prior to soft palate repair.^15,16^ The preoperative model included cleft width and presence of syndromes as key predictors for speech-correcting surgery. For the postoperative model oronasal fistula was additionally included. Both models showed moderate discriminative ability (AUROC), reflecting the complexity of predicting speech outcomes and speech-correcting surgery. Calibration of the average predicted probabilities of the models was good, though the postoperative model slightly underestimated risk in some ranges.

### Predictive Variables

Cleft width was the most significant predictor for speech-correcting surgery, with each millimeter increase in width raising the likelihood of requiring speech-correcting surgery by approximately 27–29%. A 10 mm cleft width threshold is proposed, distinguishing between narrow and wide clefts based on the average cleft width in this study (10.9 mm). Patients with cleft widths of ≥10 mm were significantly more likely to require speech-correcting surgery when compared to patients with cleft widths of <10 mm (*p* < .001).

The presence of additional syndromes and oronasal fistula, further increase chances of requiring speech-correcting surgery. Patients with additional syndromes face increased anatomical and functional complexity which can negatively impact speech outcomes. In line with previous studies, our cohort showed higher rates of speech-correcting surgery among these patients, highlighting the clinical relevance of this increased complexity.^17^ Similarly, the presence of oronasal fistula significantly increases the chances of requiring speech-correcting surgery, with a 2.18 times greater odds of requiring speech-correcting surgery in patients with fistulas. Therefore, careful management to prevent fistula formation is crucial.

Although variables such as cleft type and age at palatoplasty were not significant predictors in this study, they remain clinically relevant. Previous studies have associated more severe cleft types (i.e., higher Veau classifications) with increased speech-correcting surgery rates, although these studies often did not account for cleft width.^13^ Moreover, it has been demonstrated that higher Veau classifications are typically characterized by increased cleft width.^18^ Our findings suggest that initial cleft width is a stronger predictor of speech-correcting surgery than cleft type, as wider clefts are more challenging to close and may increase the risk of fistula formation and VPI, thereby raising the likelihood of subsequent speech-correcting surgery, a conclusion supported by prior research.^19,20^ In our cohort, patients with wider clefts (≥10 mm) showed a higher incidence of fistula, further supporting this association. In wider clefts, advanced surgical techniques like adding buccal flaps may be recommended to minimize risks such as oronasal fistula by reducing tension on the repair site.^21,22^

Postoperative complications, such as palatal dehiscence, can lead to secondary healing, scarring, or permanent oronasal fistula, potentially resulting in VPI and impaired speech and resonance.^7,23,24^ Fistula formation is primarily attributed to tension and impaired wound healing, which can shorten the palate and compromise velopharyngeal closure.^7^ Detailed early wound healing data (<6 weeks postoperative) were lacking, hence subtle scarring and secondary healing may have contributed to a shortened palate. The incidence of fistula varies widely, with meta-analyses estimating 6.4–8.6%.^25,26^ In our previous study, patients with fistulas underwent significantly more secondary surgeries (70% vs. 45%, *p* = .004), indicating a strong association.^12^ Notably, we did not differ between patients who had fistula closure prior to speech-correcting surgery and those who had it performed concurrently. Fistula closure performed before speech-correcting surgery was indicated for symptomatic air leakage, whereas in those who had fistula closure concurrently with speech-correcting surgery, the indication was insufficient velopharyngeal function resulting from a short palate, rather than nasal air emission caused by air leakage through the fistula.

### Strength and Limitations

One of the strengths of this study is that it only had few missing data and a long mean follow-up duration of ten years, providing insight on long-term outcomes. In addition, the methodology used for developing the model ensures that performance is not overoptimistic, providing robust models and enhancing their generalizability.

To fully appreciate the findings in this study, several considerations are important. First, the models showed only moderate ability in predicting speech-correcting surgery. As a retrospective cohort study, it relied on existing records and additional variables, such as more detailed surgical data, could have improved model performance. Postoperative follow-up extended to 5 years of age, which is relatively long; however, VPI may develop after adenoidal regression, around 7 years of age, and additional cases of VPI may emerge with continued follow-up. Ultimately, as a single-center study, the findings are not generalizable. The models’ performance in different clinical settings was not evaluated, and further prospective external validation is needed to assess their consistency across different cleft populations and cleft teams.

Uncertainty remains regarding the predicted probabilities provided by the models at the individual patient level. For example, a given patient might be assigned a predicted 50% probability of speech-correcting surgery by the model, but the true probability may lie between 35% and 55%. This imprecision is particularly concerning for predicted probabilities below 25% or above 70%, as reflected by the wide confidence intervals in Figures 1 and 2. While these predictions are not absolute, they do offer valuable insights into whether a patient falls into a higher or lower risk category.

### Further Research

For further research, it is crucial to document not only cleft type but also cleft width, particularly the cleft width-to-palatal-shelf ratio and total palatal length.^27^ In 2-stage surgeries, cleft width should ideally be measured both before and after vomerplasty, as the vomer flap can significantly reduce the cleft width.^15,16^ Standardized intraoperative measurements using calipers or 3D intraoral scanners, both pre- and postvomerplasty, would provide a more accurate description of cleft anatomy. Intraoral scanners, already successfully integrated in orthodontics, could similarly support a more individualized therapeutic approach.^28^ Moreover, future studies should consider whether the surgical technique chosen is appropriate for the patient, as anatomical characteristics such as cleft width may influence the suitability and outcomes of different repair methods. Prospective data collection is currently ongoing in our center, aiming to develop a more accurate prediction model for tailored surgical planning.

The finding that oronasal fistula is a positive predictor of impaired speech underscores the importance of surgical precision in preventing fistula formation, possibly adding tissue in wide clefts (i.e., buccal flaps), as well as the need for meticulous postoperative management.^29^ This includes careful dietary precautions, infection prevention (e.g., avoiding surgery during periods of illness or viral infections), and close monitoring of wound healing.^30^

The models developed in this study provide a tool for clinicians in our center to better counsel patients and parents about the probability of requiring additional speech-correcting surgery to address VPI based on cleft width, the presence of additional syndromes and oronasal fistula. Due to their moderate predictive ability, they should be used alongside, not instead of, clinical judgment. The calibration plot shows a generally good fit, however uncertainty increases at the extremes (i.e., lowest and highest predicted probabilities). Future research will focus on expanding and externally validating the models, ideally across multiple cleft centers, to improve generalizability and ensure its broader clinical utility.

## Conclusion

This study presents a prediction model for patients with CP ± L, identifying wide cleft, presence of syndromes, and oronasal fistula as key predictors for additional speech-correcting surgery. While the models demonstrate moderate predictive accuracy, they should serve as a complement to and not as replacement for clinical judgment. By offering a tool for risk assessment, this model supports more personalized consultation and tailored follow-up strategies.

## Supplemental Material

sj-docx-1-cpc-10.1177_10556656251401507 - Supplemental material for Prediction of Speech-Correcting Surgery in Patients With a Cleft Palate After Primary Palatoplasty: A Logistic Regression Model Supplemental material, sj-docx-1-cpc-10.1177_10556656251401507 for Prediction of Speech-Correcting Surgery in Patients With a Cleft Palate After Primary Palatoplasty: A Logistic Regression Model by Lieke Hofman, Kevin Jenniskens, Harm Winters, Aebele B. Mink van der Molen and Emma C. Paes in The Cleft Palate Craniofacial Journal
